# Downlink Performance Modeling and Evaluation of Batteryless Low Power BLE Node

**DOI:** 10.3390/s22082841

**Published:** 2022-04-07

**Authors:** Ashish Kumar Sultania, Carmen Delgado, Chris Blondia, Jeroen Famaey

**Affiliations:** 1 IDLab-Department of Computer Science, University of Antwerp-imec, 2000 Antwerp, Belgium; ashishkumar.sultania@uantwerpen.be (A.K.S.); chris.blondia@uantwerpen.be (C.B.); 2 i2CAT Foundation, 08034 Barcelona, Spain; carmen.delgado@i2cat.net

**Keywords:** batteryless, Bluetooth Low Energy, energy harvesting, IoT, low power node

## Abstract

Deploying low maintenance and long-life systems is an important requirement of emerging commercial Internet of Things (IoT) solutions. Such systems can be envisioned in which the connected devices are powered by energy harvested from ambient sources and stored in long-lifetime capacitors rather than short-lived and polluting batteries. However, due to the unpredictable nature of ambient energy harvesting, such batteryless IoT devices might not always have enough energy to initiate communication. The Bluetooth Low Energy (BLE) specification defines support for Low Power Nodes (LPNs) using the friendship feature, where the LPN is associated with a neighbouring friend node (FN). The LPN can receive downlink (DL) data and remain connected to the network via the FN that buffers the LPN’s incoming packets while allowing the LPN to save energy by sleeping or turning itself off. This novel BLE feature makes the LPN highly suitable to support the connection of batteryless ambiently-powered IoT devices. While the LPN can decide when to transmit uplink (UL) packets and does not depend on the FN to receive downlink (DL) data, the LPN needs to poll its FN to receive the buffered packets. However, the DL packet latency increases with this process due to the buffering time at the FN. Therefore, in this work, we present an analytical model to characterize the performance as a function of DL data latency and packet delivery ratio (PDR) of a batteryless LPN powered by different harvesting powers and capacitor sizes. This would help to optimally choose the correct configuration of the batteryless LPN for its network deployment. We also compare the analytical model and simulation results, showing consistency with an average error of 2.23% for DL data latency and 0.09% for the PDR.

## 1. Introduction

The emerging Internet of Things (IoT) concept aims to wirelessly connect billions of devices to the internet. Usually, these devices are powered by batteries. However, replacing batteries for such a large number of devices increases the cost of system maintenance, as it requires manual intervention. Battery life is also reduced due to over-charging and over-discharging, as well as environmental conditions (e.g., temperature fluctuations) [1]. Moreover, disposing of batteries is also a serious environmental concern. Therefore, considering the downside of battery usage and the requirement of low maintenance ’deploy and forget’ devices for the IoT, batteries can be replaced by easily recyclable and cheap capacitors combined with energy harvesting to obtain and store energy in a more sustainable way [2,3]. Such devices can achieve a long-term multi-decade active lifetime.

Bluetooth Low Energy (BLE) has become one of the most important technologies for the IoT thanks to its features (i.e., extremely low power consumption, low cost, and mesh topology support). It supports many use-cases, such as asset tracking, sensing, monitoring, control, and automation, with thousands of interconnected devices [4]. However, most existing BLE-based IoT solutions consider battery-powered devices. Meanwhile, designing a batteryless solution is important for long-term sustainability (i.e., no adverse impact on the environment), but it is also a challenging task. Batteryless devices usually charge their capacitor by harvesting ambient energy, which is often uncontrollable and unpredictable, allowing them to only work for a short amount of time. These conditions can result in intermittent behaviour where the device can experience multiple power failures. The duration of the power failure cycle depends on the harvesting power, capacitor size, and operating voltage. Moreover, for a specific harvesting power, the time to charge a capacitor increases with its capacitance [5]. Therefore, choosing the optimal capacitor size based on the minimum available harvesting power and required operations to achieve the desired performance is important.

The BLE specification [6] already provides a friendship feature to aid Low Power Nodes (LPNs) to save energy, keeping themselves in SLEEP or OFF mode for most of the time. The LPN awakes only (or turns on) to transmit or receive data packets. For sending uplink (UL) data packets, the LPN can broadcast them at any time. On the other hand, the LPN can start a poll process (request/response) to receive the downlink (DL) packets that are temporarily buffered at the friend node (FN). The polling happens periodically to receive any incoming data packets. This can reduce the overall energy consumption at the cost of increased DL data latency.

In battery-powered nodes (non-harvesting), long-term energy consumption is a key factor as it affects the total lifetime of the device. This factor is less important in batteryless devices powered by ambient energy as they can perpetually recharge themselves. Instead, short-term energy consumption bursts are more important as they may drain the capacitor and result in a power failure. In this article, we focus on analyzing the achievable DL latency and packet delivery ratio (PDR) using a batteryless BLE LPN supported by different harvesting powers and capacitor sizes. We model the system utilizing a discrete-time Markov Chain and compare the results with our developed simulator [7]. The simulator needs to simulate extended periods of time and be repeated many times to average out stochastic variability, whereas a model can immediately provide the results for a specific use case at steady state. This allows the model, compared to a full-fledged simulator, to be integrated into a real-time optimizer in the node that can help to easily reconfigure the BLE configuration parameters based on current conditions. The model can be used to theoretically perform a feasibility analysis of the friendship feature for bidirectional battery-less communication and to deduce its limitations. Moreover, it would aid in designing battery-less BLE devices by calculating its optimal hardware and software configuration for IoT use-cases that require downlink or bidirectional communication. Specifically, our model can be used to optimally derive the hardware configuration in terms of capacitor size. The software configuration parameters, in terms of ReceiveDelay, ReceiveWindow, Tx power level, and Poll interval of the batteryless LPN can be determined based on the use-case requirement in terms of data rate, DL latency, and PDR considering the neighboring FN queue size and minimum harvesting power at the network site.

The paper is organized as follows: Section 2 summarizes related work; Section 3 gives an overview of the BLE friendship feature; Section 4 explains the batteryless LPN system; and Section 5 presents the analytical model. Subsequently, Section 6 presents all the evaluation results and Section 7 shows the usage of model to derive optimal parameters by considering a logistic management IoT use-case. Section 8 draws the main conclusions. Finally, Section 9 briefly discusses limitations of the model and potential avenues for future work.

## 2. Related Work

There are several batteryless BLE prototypes designed by researchers. Silva et al. [8] presented a prototype where an implantable batteryless glucose monitor node was capable of transmitting data to a mobile phone when supplied power wirelessly using a Radio Frequency (RF) energy source. However, their design did not consider the Bluetooth mesh friendship feature and sent only UL data. Liu et al. recently proposed another RF-power based prototype for indoor BLE beaconing [9]. They tested the device for both UL and DL data. However, they did not analyze the effect of the capacitor size nor the performance in terms of DL latency. Fraternali et al. [10] designed a batteryless BLE sensor node based on ambient light energy harvesting. They presented a predictive algorithm that adapted to the changing ambient conditions to maximize the node lifetime and application quality of service. However, their focus was also only on the UL data. Nilsson et al. also conducted indoor and outdoor experiments to evaluate power consumption, range, throughput, and UL latency of a Bluetooth mesh network [11]. In summary, most of the research on batteryless BLE nodes is performed considering UL data without considering the friendship feature or DL communications.

There are two papers that considered the friendship feature. However, they considered battery-powered LPNs rather than batteryless LPNs. The first paper is by Alvarez et al. [12], who performed an in-depth analysis of security on the friendship concept. They demonstrated that a denial-of-friendship attack could reduce the LPN’s battery life by 70-fold. The second paper is by Darroudi et al. [13]. They presented an analytical model to study the energy performance parameters of a battery-operated BLE LPN. These parameters include current consumption, lifetime, and energy consumed per delivered bit for different PollTimeout and ReceiveWindow values. They calculated the evaluation parameters based on the average value of time and current consumption for all the radio states and for fixed UL data rate interval, in contrast to DL traffic, which does not require the polling feature or friend queue.

In contrast, our work is focused on the DL latency and packet delivery metrics. We focus on a batteryless LPN harvesting energy from ambient sources and not on battery power LPNs. As such, we do not consider current consumption as the evaluation metric, but rather a constraint that should be satisfied. Moreover, we focus on DL packet arrivals, which are first queued at the FN, and so our model studies the queueing performance based on the Markov chain with Poisson distributed data arrivals which provides discrete random distribution of data. In real-world applications, the data arrivals are also random, and so it is used to represent the message arrivals in the BLE network. The model provides the opportunity to analyze the batteryless LPN powered by different capacitor sizes receiving different harvested powers for various Bluetooth mesh network parameters, such as friend queue size, packet size, or receive delay. The model is also compared to our developed simulator as presented in [7].

## 3. BLE Friendship Feature

**Figure 1:**
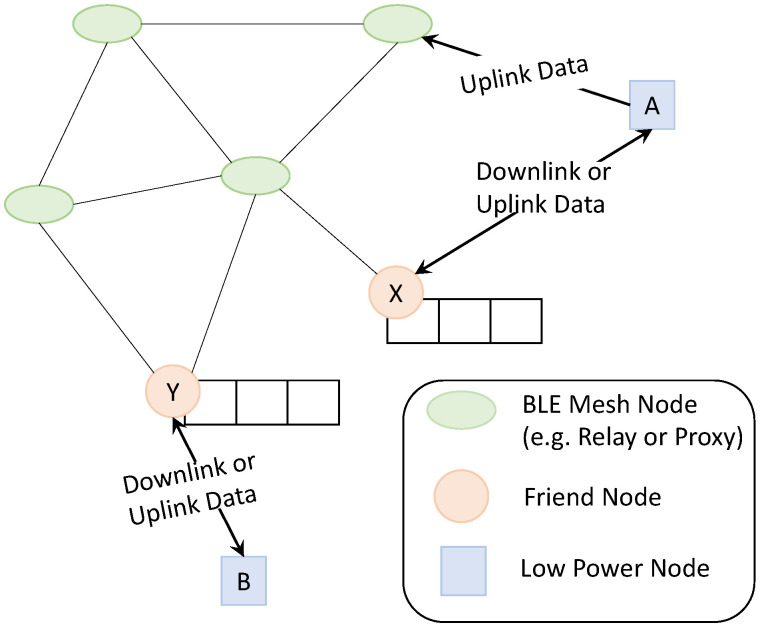
BLE mesh network.

The Bluetooth mesh network is based on a publish-subscribe model for data communication. It supports up to 32,767 devices with a maximum of 128 hops [14]. The devices need to have addresses and security information to become part of the mesh network to send or receive data. This is enabled by a provisioner, and the provisioned device is called anode. Any node can support four additional optional features (namely, relay, proxy, friend, and low power [6]), as shown in Figure 1.

The relay node supports receiving and re-transmitting data packets to all the nodes in its connection range. It helps to extend the range of the entire network. The proxy node helps the non-mesh-supported BLE devices to communicate via the mesh network. To enable both these features, the node needs to listen continuously for advertising packets and, therefore, should be connected to a continuous power source [15]. To support energy-constraint nodes, BLE introduces the LPN, which can offload the task of listening to its incoming channel to a FN that can temporarily buffer any incoming data packets. For example, in Figure 1, LPN ‘A’ is associated with FN ‘X’ for the DL data. However, it does not need help to transmit UL data. The LPN can wake up any time to send UL data, assuming that at least one of its neighbouring nodes actively listens to the incoming channels. With the help of its FN, the LPN can save much of its energy by turning off its components and keeping itself in SLEEP mode for most of the time. However, before switching to the SLEEP or OFF state, the LPN needs to first establish a friendship with one of its single-hop neighbouring nodes. This neighbour needs to support the optional friendship feature.

### 3.1. Friendship Establishment

**Figure 2:**
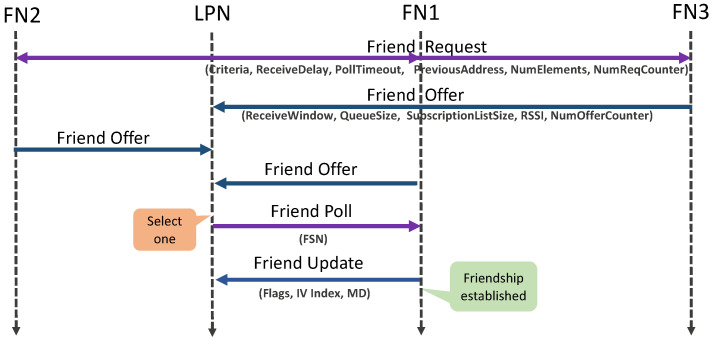
Friendship establishment procedure of BLE.

Figure 2 illustrates the messages exchanged during the friendship establishment procedure. The same Friend Request is received by one or more neighbouring friend feature enabled nodes. This message is 28 bytes of a network Protocol Data Unit (PDU), which consists of Criteria, ReceiveDelay (RD), PollTimeout (PT), PreviousAddress, NumElements, and NumReqCounter [6].

**Figure 3:**
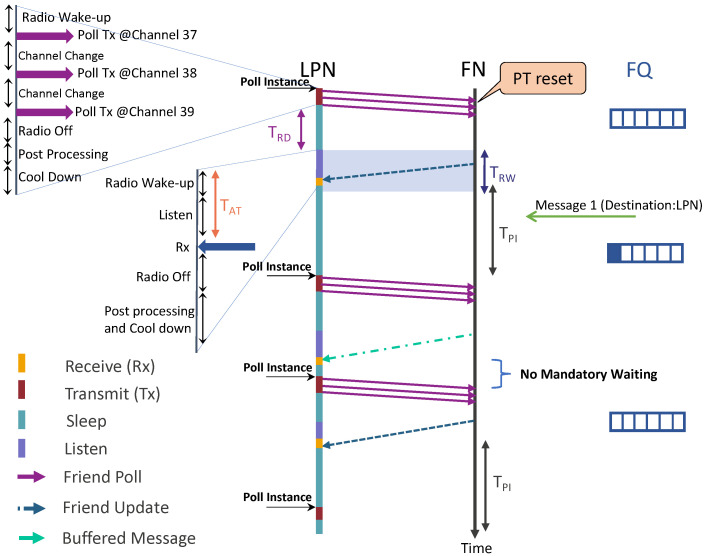
Sequence diagram of the messages exchanged between the LPN and FN to receive DL packets.

The Criteria is the minimum requirement in terms of the Received Signal Strength Indicator (RSSI), ReceiveWindow (RW), and supported queue size that an FN should satisfy. The RD is the waiting time of the LPN before it should expect to receive a poll response from the FN. PT is used to ensure that the FN does not keep the friendship context indefinitely and terminates the relationship if it does not receive any poll before the PT timer expires (since the last poll). Lastly, the RW is the maximum time that the LPN should listen to receive a response from the FN after sending a poll request. These timers can be seen in Figure 3. The RD and RW can be configured with a maximum value of 255 ms and a PT with a maximum of 96 hours. PreviousAddress defines the address of its previous FN, NumElements defines its number of addressable elements (as messages in the BLE mesh network are exchanged from the elements), and NumReqCounter defines the number of sent requests required to calculate the Friendship Security Material.

The FNs that satisfy the initial criteria defined by the LPN will offer the friendship by sending a Friend Offer (FO). This response message has a network PDU of 24 bytes, which consists of RW, supported friend queue (FQ) size, size of subscription list, calculated RSSI, and number of FO sent by this FN. Based on all the received FOs, the LPN selects the best among them, considering the Criteria satisfactions. To confirm this selection, the LPN acknowledges the selected FN with a Friend Poll (FP) message of 19 bytes, with the Friend Sequence Number (FSN) equal to zero. Next, the FN finalizes the friendship procedure by sending a Friend Update (FU) message of 24 bytes that contains the flags, More Data (MD), indicating if the queue is empty or not, and another flag that indicates the update of the initialization vector (IV) index (which is a shared network resource) or security key. It also contains the current IV index value known by the FN. Once the friendship is established, the LPN also notifies the FN with all the subscribed group list addresses so that the FN knows which messages it should buffer and forward to the LPN.

### 3.2. Receiving Downlink Data

As the friendship is established, the LPN can now switch to SLEEP mode, and all the incoming DL packets of the LPN will be buffered in the FQ. The process is shown in Figure 3.

The LPN can retrieve them from the FN by periodically waking up and sending an FP message in all three broadcast channels (37 to 39). On reception of the FP, the FN gets a time window of RW ($T_{RW}$) seconds, starting ($T_{RD}$) seconds after receiving the FP to send a response [16]. We also define an arrival time (AT or $T_{AT}$), which denotes the total listening time until the LPN starts receiving the message. While sending the FP, the LPN needs to toggle the FSN value to indicate the acknowledgement of the previous message. If the FN receives the same FSN field value as the last FP message received from the FN, it responds with the same message that it has previously sent, unless the FQ head is updated. This can be updated by any new incoming packet(s) whenever it arrives at a full FQ. In this case, the oldest packet (FQ head) would be discarded (dequeued) and the newly arrived packet would be added in the queue tail (queued). This strategy is called ‘push-out’. Therefore, if the previously sent message has been discarded (on the arrival of new packets), then the oldest entry in the FQ shall be sent. If the FQ is empty, an FU is sent with MD equal to zero. To avoid receiving frequent, continuous FU messages, a timer poll interval timer ($T_{PI}$) can be considered. On receiving an FU message, an LPN needs to wait until the PI expires to send the next FP message. As we consider a batteryless LPN, it may happen that it is unable to send the FP exactly at the expiration of PI due to energy constraints. The friendship can also be terminated if the LPN does not receive any response from the FN after sending the FP for a certain amount of attempts. Moreover, BLE mesh provides infrastructure-less connectivity without any overheads of time synchronization [17]. As the nodes (except LPNs) are assumed to be always listening, there is no need to synchronize their listening timeslots. Moreover, the LPN always initiates communication with the FN itself (i.e., receiver-initiated communication); therefore, the LPN does not need to synchronize explicitly for receiving data either [16]. Timing of the RW is done relative to the FP message sent by the LPN to its FN.

## 4. Batteryless BLE LPN

Let the LPN have an already established friendship with a neighbouring FN. To receive DL packets, the radio of an LPN can be in 15 different states, as shown in Figure 3 (radio wake-up to post-processing and cool down). The voltage (energy) of a capacitor as a function of time, supporting various states of the radio as used in the remainder of this section, was first introduced by Delgado et al. [18].

### 4.1. Capacitor Voltage

Equation (1) provides the voltage on the capacitor after the device operates at a load current of $I_{L}\left(X\right)$ (amperes) in state X for a time Δt, harvesting from a constant voltage source, providing a power equal to $P_{h}$ (watts).

(1) $$\begin{array}{cc} \; & V\left(t+\Delta t\right)=\frac{V_{max}\cdot P_{h}}{P_{h}+V_{max}I_{L}\left(X\right)}\left(1-e^{\frac{-\Delta t\cdot (P_{h}+V_{max}I_{L}\left(X\right))}{V_{max}^{2}\cdot C}}\right)+V\left(t\right)\cdot e^{\frac{-\Delta t\cdot (P_{h}+V_{max}I_{L}\left(X\right))}{V_{max}^{2}\cdot C}}, \end{array}$$

where, $V_{max}$ (volt) is the maximum capacitor voltage for capacitance C (farad).

**Figure 4:**
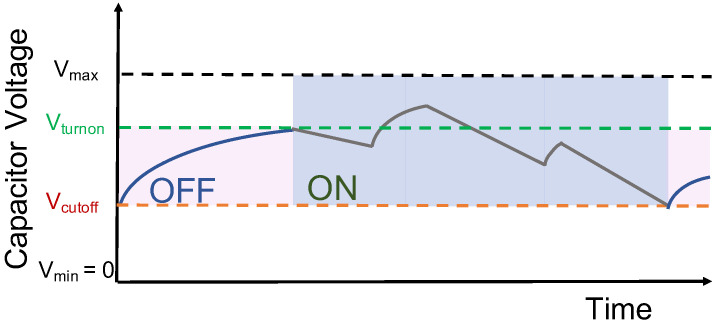
Intermittent behaviour of a batteryless LPN harvesting continuously.

As the capacitor has a small energy density and due to the unpredictable availability of harvested energy, it can result in intermittent on-off behaviour of the batteryless LPN, as shown in Figure 4. When the capacitor voltage drops below the minimum operating voltage ($V_{cutoff}$), the LPN transitions to the OFF state. If the LPN is in the OFF state, it transitions to the SLEEP state only when achieving a predefined turn-on voltage $V_{turnon}$.

However, in our model, an energy-aware LPN is considered that tries to avoid the OFF state. It can use an ultra-low-power comparator, with power consumption in the order of pico-watts [19], which can determine if a defined voltage level in the capacitor has been reached. This voltage could be set to the minimum voltage level required to execute a task (such as poll, receive data) without going below $V_{cutoff}$. We refer to that approach as an energy-aware device.

As stated before, the friendship can be terminated if no response is received either after sending $\dot{N}$ FPs or if the time between two consecutive FPs is larger than $T_{PT}$. To avoid this, the $P_{h}$ is considered such that it allows the capacitor to recharge itself from $V_{min}$ to a threshold voltage level that is sufficient to perform a poll request-response cycle in less than $T_{PT}$. The time to send the FP when the batteryless LPN needs to have a required threshold voltage of $V_{th}^{FP}$ to start the FP and the remaining voltage of the capacitor after an FP is calculated in Section 4.2.

### 4.2. Time to Next Poll

The time at which the next FP can take place depends on whether the FQ is empty or not and the available energy after the previous poll.

#### 4.2.1. FQ Is Not Empty

The voltage of the capacitor after all the polling stages can be calculated according to Equation (1), starting from an initial voltage ($V_{0}$) and considering ‘radio wake-up’ as an initial state. Let $V_{f}$ be the final voltage after all the polling radio stages (as shown in Figure 3) of the previous poll are executed. Therefore, there are two scenarios:

- $V_{f}\geq V_{th}^{FP}$: The next FP can be sent immediately, and the time between two FPs ($T_{FP}$) is the sum of all the state timings as defined in Table 1 and is calculated as Equation (2). The capacitor voltage at the start of the next poll instance will be $V_{f}$.
  (2) $$\begin{array}{c} T_{FP}=\sum_{i=AllStates}T_{i} \end{array}$$
- $V_{f}<V_{th}^{FP}$: As $V_{f}$ is less than the required voltage to poll, the device needs to switch to SLEEP mode to charge the capacitor and avoid its voltage from reaching $V_{cutoff}$. Let the node sleep for the time $T_{sleep}$ which can be deduced using Equation (1). Therefore, the time between two FPs has an extra wait time of $T_{sleep}$ in addition to all the FP state’s timings and is calculated in Equation (3).
  (3) $$\begin{array}{c} T_{FP}=\sum_{i=AllStates}T_{i}+T_{sleep} \end{array}$$
  The capacitor voltage at the start of the next poll instance will be $V_{th}^{FP}$ (after the capacitor is charged while the LPN is in SLEEP mode).

#### 4.2.2. FQ Is Empty

If the FQ is empty, the LPN needs to wait for the time PI ($T_{PI}$) to poll, as it received an FU after the previous FP message. Therefore, in this case, the voltage $V_{f}$ would be calculated after a sleep time of $T_{PI}$. Now again, there are two cases.

- $V_{f}\geq V_{th}^{FP}$: The time to poll has an extra delay of $T_{PI}$ in addition to all the FP state’s timings.
  (4) $$\begin{array}{c} T_{FP}=\sum_{i=AllStates}T_{i}+T_{PI} \end{array}$$
  The capacitor voltage at the start of the next poll instance will be $V_{f}$.
- $V_{f}<V_{th}^{FP}$: Similar to above, the node needs to switch to SLEEP mode to provide enough time to charge the capacitor. Therefore, the time between two FPs has an additional time $T_{sleep}$ and $T_{PI}$ in addition to all the FP state’s timings.
  (5) $$\begin{array}{c} T_{FP}=\sum_{i=AllStates}T_{i}+T_{PI}+T_{sleep} \end{array}$$
  The capacitor voltage at the start of the next poll instance is $V_{th}^{FP}$.

The threshold voltage of the FP ($V_{th}^{FP}$) can be calculated by taking the inverse of the function, as mentioned in Equation (1) calculating *V*(*t*), knowing the value of V(t+Δt) for all the states of the poll, considering the initial voltage as $V_{cutoff}$, and with starting state ‘post-processing and cool down’.

## 5. System Model

This section presents the analytical model of the system described in Section 4. The DL latency, FQ occupancy, and PDR are calculated. All the symbols used in this section are summarized in Appendix A.

### 5.1. The Queueing Model

We call the time interval between two FPs, when the FQ at the start of the interval is not empty, a ‘service’. The time interval between two FPs, during which an FU is transmitted from the FN to the LPN (i.e., the FQ at the start of the interval is empty), is called a ‘vacation’. The system can be modelled as a finite capacity queue, where the server takes repeated vacations. The duration of a service and a vacation depends on the LPN’s capacitor voltage. Let the packets arrive at the FN according to a Poisson distribution with data arrival rate λ, and the FN temporarily buffers them in a buffer (FQ) of capacity *N*. The vector U represents the discretized voltages that a capacitor can take between 1 and $V_{max}$. Hence, the duration of a service when the voltage equals i∈U can be denoted by $S_{i}$ with distribution function $S_{i}\left(t\right)$ and Laplace Transform (LST) $S_{i}^{*}\left(\nu \right)$. Similarly, for the vacation, $W_{i}$, $W_{i}\left(t\right)$ and $W_{i}^{*}\left(\nu \right)$ can represent the duration, the distribution function, and its LST, respectively. The duration of a service consists of all the radio states during polling and possibly followed by a sleep period to reach the threshold index $V_{th}^{FP}$. The radio states during the polling can be assumed to have a constant duration for a fixed size of the DL packet. Hence, $S_{i}$ can be computed considering FQ is not empty and $W_{i}$ considering it is empty, as discussed in Section 4.2. If at the start of a service the voltage is equal to i, then the voltage at the next poll instance is denoted by $V_{i}^{S}$ and calculated using Equation (1) for all the polling radio states. Similarly, if the voltage is equal to i at the start of a vacation, its values at the next inspection instant denoted by $V_{i}^{W}$ also include the SLEEP state for $T_{PI}$.

The probability $P_{k,i}^{S}$ and $P_{k,i}^{W}$ that k packets arrive during the service time and the vacation time respectively, starting from U, equal to i, can be calculated as Equation (6).

(6) $$\begin{array}{c} P_{k,i}^{S}=\int_{0}^{\infty }\frac{{\left(\lambda t\right)}^{k}}{k!}e^{-\lambda t}dS_{i}\left(t\right)\\ P_{k,i}^{W}=\int_{0}^{\infty }\frac{{\left(\lambda t\right)}^{k}}{k!}e^{-\lambda t}dW_{i}\left(t\right) \end{array}$$

The column vectors of the averages of the service times and the vacation times are given by Equation (7).

(7) $$\begin{array}{c} \bar{E[S]}={\left(E\left[S_{1}\right],E\left[S_{2}\right],\ldots ,E\left[S_{V_{max}}\right]\right)}^{{}^{\prime }}\\ \bar{E[W]}={\left(E\left[W_{1}\right],E\left[W_{2}\right],\ldots ,E\left[W_{V_{max}}\right]\right)}^{{}^{\prime }} \end{array}.$$

### 5.2. Embedded Markov Chain

The system is observed at the poll instants ($t_{n}$, *n* = 1, 2, ⋯, *∞*). We refer to these poll instants as inspectioninstants. Let $Q_{n}$ be the number of packets in the FQ and $V_{n}$ be the available capacitor voltage at time instant $t_{n}$. It should be noted that $V_{n}$ will never take values below $V_{startpoll}$, which is the minimum voltage available at a poll instant that allows all functions of the radio to receive a packet. Hence, the stochastic process ($Q_{n},V_{n}$) at $t_{n}$ is a discrete-time finite Markov Chain with state-space size equal to $\left(N+1\right)\cdot (V_{max}-V_{startpoll}+1)$. The limiting probability distribution function of this Markov Chain is denoted by $\bar{p}=\left({\bar{p}}_{0},{\bar{p}}_{1},\ldots ,\bar{p_{N}}\right)$. Let (${\bar{p}}_{k}{)}_{i}$ denote the joint probability distribution of the voltage and the queue length, as shown in Equation (8).

(8) $$\begin{array}{cc} {\left({\bar{p}}_{k}\right)}_{i} & =\lim_{n\to \infty }Prob\left(Q_{n}=k,V_{n}=i\right),\\ & \;\;\;\;\mathrm{where},\;0\;\leq k\leq N,i=V_{startpoll},\ldots V_{max} \end{array}$$

The transition matrix of this Markov Chain is given by Equation (9).

(9) $$P=[\begin{array}{ccccccc} B_{0} & B_{1} & B_{2} & \ldots & B_{N-2} & B_{N-1} & \sum_{n=N}^{\infty }B_{n}\\ A_{0} & A_{1} & A_{2} & \ldots & A_{N-2} & \sum_{n=N-1}^{\infty }A_{n} & 0\\ 0 & A_{0} & A_{1} & \ldots & A_{N-3} & \sum_{n=N-2}^{\infty }A_{n} & 0\\ \vdots & \vdots & \vdots & \vdots & \vdots & \vdots & \vdots \\ 0 & 0 & 0 & \ldots & A_{0} & \sum_{n=1}^{\infty }A_{n} & 0\\ 0 & 0 & 0 & \ldots & 0 & \sum_{n=0}^{\infty }A_{n} & 0 \end{array}]$$

where, $B_{n}$ represents the transitions starting from an empty system and ending up with *n* packets in the FQ at the next inspection point. Whereas, $A_{n}$ represents the transitions starting from a non-empty system. The ($V_{max}-V_{startpoll}+1)\cdot \left(V_{max}-V_{startpoll}+1\right)$ size-matrices $A_{n}$ are defined by Equation (10).

(10) $${\left(A_{n}\right)}_{i,j}=\left\{\begin{array}{c} P_{n,i}^{S},\;\;\mathrm{for}\;i=\;[V_{startpoll},V_{max}],\;j=V_{i}^{S}\;\\ 0,\;\mathrm{elsewhere} \end{array}\right.$$

Similarly, $B_{n}$ can also be calculated as Equation (11).

(11) $${\left(B_{n}\right)}_{i,j}=\left\{\begin{array}{c} P_{n,i}^{W},\;\mathrm{for}\;i=\;[V_{startpoll},\;V_{max}],\;j=V_{i}^{W}\\ 0,\mathrm{elsewhere} \end{array}\right.$$

In the numerical examples, for a large value of k, $P^{k}$ has positive real entries. Therefore, the matrix P is regular and has a unique normalized eigenvector $\bar{p}$ corresponding to the eigenvalue 1. Hence, the probability distribution satisfies Equation (12).

(12) $$\begin{array}{cc} \; & \bar{p}=\bar{p}\cdot P\\ & \bar{p}\cdot \bar{e}=1\;,\\ & \mathrm{where},\;\bar{e}\;\mathrm{is}\;\mathrm{the}\;\left(N+1\right)\cdot (V_{max}-V_{startpoll}+1)\;\mathrm{unit}\;\mathrm{vector}. \end{array}$$

The steady-state probability of the system occupancy at arbitrary time instants has been derived in [20]. Let $T_{\mathrm{FP}}$ be the average time between two consecutive inspection instants given by Equation (13).

(13) $$\begin{array}{c} T_{FP}=\left[1-{\bar{p}}_{0}\cdot \bar{e}\right]\cdot E\left[S\right]+{\bar{p}}_{0}\cdot \bar{e}\cdot E\left[W\right], \end{array}$$

where

(14) $$E\left[S\right]=\left\{\begin{array}{c} \frac{\sum_{n=1}^{N}{\bar{p}}_{n}\cdot \bar{E[S]}}{1-\bar{p_{0}}\cdot \bar{e}},\;if\;\bar{p_{0}}\cdot \bar{e}\neq 1\\ 0,\;\;\;\;\;\;\;\;\;\;\;\;if\;\bar{p_{0}}\cdot \bar{e}=1\;\left(system\;is\;always\;empty\right) \end{array}\right.$$

and

(15) $$E\left[W\right]=\left\{\begin{array}{c} \frac{\bar{p_{0}}\cdot \bar{E[W]}}{\bar{p_{0}}\cdot \bar{e}},\;if\;\bar{p_{0}}\cdot \bar{e}\neq 0\\ 0,\;\;\;\;\;\;\;\;\;\;\;\;\;\;\;\;if\;\bar{p_{0}}\cdot \bar{e}=0\;\left(system\;is\;never\;empty\right) \end{array}\right.$$

The queue occupancy distribution, i.e., the probability that there are n packets in the FQ, is then given by Equation (16) [20].

(16) $$\begin{array}{cc} \; & O_{n}=\frac{1}{\lambda \cdot T_{FP}}\cdot \left[{\bar{p}}_{n}\cdot \bar{e}-{\bar{p}}_{0}\cdot B_{n}\cdot \bar{e}\right],\;\;\;\;\;\;\;\;\mathrm{where},\;n\;=\;[0,\;N-1]\\ & \mathrm{And}\\ & O_{N}=1-\frac{1}{\lambda \cdot T_{FP}}\cdot \left[1-{\bar{p}}_{0}\cdot \bar{e}\right]\;. \end{array}$$

Therefore, the total percentage of packet loss is equal to $100\cdot O_{N}$.

### 5.3. Performance Metrics

In this section, the metrics, average FQ occupancy, and average DL latency, are computed.

- Average FQ occupancy: The FN uses a push-out strategy to manage its FQ, which means when a packet arrives at a full FQ, the oldest packet in the FQ would be dropped. However, the number of packets in the *push-out* system is the same even if the arriving packet is dropped (drop-tail). Since the probability that an arriving packet finds a full FQ is equal to $O_{N}$, the average system occupancy in a ‘drop-tail’ system is given by Equation (17).
  (17) $$\begin{array}{c} O_{DT}=\sum_{n=1}^{N}n\cdot O_{n}\;. \end{array}$$
  Now, the average system occupancy in a ‘push-out’ system ($O_{PO}$) is equal to the $O_{DT}$.
- Average DL latency: The actual arrival rate in a drop-tail system is given by $(1-O_{N})\cdot \lambda$ and, applying Little’s well-known formula [21], the average response time in a drop-tail system is given by Equation (18).
  (18) $$\begin{array}{c} T_{DT}=\frac{O_{DT}}{(1-O_{N})\cdot \lambda }\;. \end{array}$$
  However, the waiting time of a packet in the system using push-out is quite different from the waiting time in a system using drop-tail. In a drop-tail system, the packets behind a tagged packet do not impact the delay of that packet. However, in a push-out system, packets arriving after the tagged packet may, in case they arrive at a full queue, push out the first packet in the queue and hence influence the delay experienced by the tagged packet. The waiting time in the push-out system consists of two parts: firstly, the time interval between the packet arrival instant (at FQ) and the next inspection instant (i.e., until the end of the vacation if it arrives during a vacation or until the end of a service if it arrives during a service); secondly, the time interval between this inspection instant and the start of the transmission of the packet.
  - Between arrival instant and the next inspection instant: There are two scenarios, either the packet arrives during the vacation period or during the service period.
    * An arbitrary time instant falls in a vacation: Let $P_{n}^{\omega }\left(t\right)$ be the probability that an arbitrary time instant falls in a vacation period, that there are n packets (maximum N) present in the system at time *t*, and that the remaining vacation time is $T_{v}$ that satisfies $t\leq T_{v}<t+dt$. The LST of this probability has already been determined by Blondia [20]. Hence, the inverse LST can provide us the value of $P_{n}^{\omega }\left(t\right)$ and $P_{N}^{\omega }\left(t\right)$, as defined in Equation (19).
      (19) $$\begin{array}{cc} \; & P_{n}^{\omega }\left(t\right)=\frac{1}{\lambda \cdot T_{FP}}\cdot [{\bar{p}}_{0}\cdot \bar{e}\cdot \mu_{E[W]}\left(t\right)\cdot {\left(-1\right)}^{n+1}\cdot e^{\lambda \cdot (t-E[W])}\cdot \frac{{\left(t-E\left[W\right]\right)}^{n}}{n!}\\ & \;\;\;\;\;\;\;\;\;-\sum_{l=0}^{n}\left({\bar{p}}_{0}\cdot B_{l}\cdot \bar{e}\cdot \lambda^{n-l+1}\cdot {\left(-1\right)}^{n-l+1}\cdot e^{\lambda \cdot t}\cdot \frac{t^{n-l}}{(n-l)!}\right)].\\ & \mathrm{and}\;\\ & P_{N}^{\omega }\left(t\right)=\frac{{\bar{p}}_{0}\cdot \bar{e}}{T_{FP}}\cdot \left[1-\mu_{E[W]}\left(t\right)\right]-\sum_{n=0}^{N-1}P_{n}^{\omega }\left(t\right), \end{array}$$
      where, *n* = [0, *N* − 1] and $\mu_{E[W]}$(*t*) is a Heaviside function that outputs (0) if *t* ≤ E[W] and (1) if *t* > E[W].
      $P_{n,k}^{\omega }\left(t\right)$ represents the probability that an arbitrary time instant falls in a vacation period, that there are n packets (maximum N) present in the system at time t, and there are k packet arrivals (maximum N−1) during the remaining vacation time $T_{v}$. This probability is given by Equation (20).
      (20) $$\begin{array}{cc} P_{n,k}^{\omega }\left(t\right) & =e^{-\lambda \cdot t}\frac{{\left(\lambda \cdot t\right)}^{k}}{k!}\cdot P_{n}^{\omega }\left(t\right)\;. \end{array}$$
      The probability that at an arbitrary time instant there are n packets (maximum N−1) in the system and that, during the remaining time of the vacation k, packets (maximum N−1) have arrived is given in Equation (21).
      (21) $$\begin{array}{cc} \; & P_{n,k}^{\Omega }=\int_{0}^{\infty }P_{n,k}^{\omega }\left(t\right)dt=\frac{1}{\lambda T_{FP}}\sum_{l=0}^{n}\left({\bar{p}}_{0}\cdot B_{l}\cdot \bar{e}\right)\cdot \left[\frac{{\left(\lambda \cdot E\left[W\right]\right)}^{n-l+k+1}\cdot {\left(-1\right)}^{n-l}}{\left(n-l)!\cdot k!\cdot (n-l+k+1\right)}\right]\\ & \mathrm{And}\;\\ & P_{N,k}^{\Omega }=\frac{{\bar{p}}_{0}\cdot \bar{e}}{\lambda T_{FP}}\cdot \left[1-e^{-\lambda \cdot E[W]}\cdot \sum_{i=0}^{k}\frac{{\left(\lambda \cdot E\left[W\right]\right)}^{i}}{(i)!}\right]-\sum_{n=0}^{N-1}P_{n,k}^{\Omega }. \end{array}$$
      The average waiting time until the next inspection instant of a packet that arrives at an arbitrary time instant in a vacation period is then given by Equation (22).
      (22) $$\begin{array}{cc} \; & T_{n,k}^{\Omega }=\int_{0}^{\infty }t\cdot P_{n,k}^{\omega }\left(t\right)dt=\frac{1}{\lambda T_{FP}}\sum_{l=0}^{n}\left({\bar{p}}_{0}\cdot B_{l}\cdot \bar{e}\right)\cdot \\ & \;\;\;\;\;\;\;\;\;\left[\frac{{\left(\lambda \cdot E\left[W\right]\right)}^{n-l+k+1}\cdot {\left(-1\right)}^{n-l}\cdot E\left[W\right]}{\left(n-l)!\cdot k!\cdot (n-l+k+2\right)}\right]\\ & \mathrm{And}\;\\ & T_{N,k}^{\Omega }=\frac{{\bar{p}}_{0}\cdot \bar{e}\cdot \left(k+1\right)}{\lambda^{2}\cdot T_{FP}}\cdot \left[1-e^{-\lambda \cdot E[W]}\cdot \sum_{i=0}^{k+1}\frac{{\left(\lambda \cdot E\left[W\right]\right)}^{i}}{(i)!}\right]-\sum_{n=0}^{N-1}T_{n,k}^{\Omega }. \end{array}$$
    An arbitrary time instant falls in a service: Let $P_{n}^{\pi }\left(t\right)$ be the probability that an arbitrary time instant falls in a service period, that there are n packets (maximum N) present in the system at time t, and that the remaining service time $T_{s}$ satisfies $t\leq T_{s}<t+dt$. Based on the Laplace transform, [20] $P_{n}^{\pi }\left(t\right)$ can be calculated as Equation (23).
      (23) $$\begin{array}{cc} \; & P_{n}^{\pi }\left(t\right)=\frac{1}{\lambda \cdot T_{FP}}\cdot [\sum_{l=1}^{n}({\bar{p}}_{1}\cdot \bar{e}\cdot \lambda^{n-l}\cdot \mu_{E[S]}\left(t\right)\cdot {\left(-1\right)}^{n-l+1}\cdot e^{\lambda \cdot (t-E[S])}\\ & \cdot \frac{{\left(t-E\left[S\right]\right)}^{n-l}}{(n-l)!})-\sum_{l=1}^{n}\left({\bar{p}}_{l-1}-{\bar{p}}_{0}\cdot B_{l-1}\cdot \bar{e}\cdot \lambda^{n-l}\cdot {\left(-1\right)}^{n-l+1}\cdot e^{-\lambda \cdot t}\cdot \frac{t^{n-l}}{(n-l)!}\right)],\\ & \mathrm{and}\;\\ & P_{N}^{\pi }\left(t\right)=\frac{\sum_{l=1}^{N-1}{\bar{p}}_{1}\cdot \bar{e}}{T_{FP}}\cdot \left[1-\mu_{E[S]}\left(t\right)\right]-\sum_{n=1}^{N-1}P_{n}^{\pi }\left(t\right).\\ & \mathrm{where},\;n\;=\;[0,\;\ldots ,\;N-1]. \end{array}$$
      The probability that a packet arrives in a service period and n packets are present in the FQ at time t and k packets arrive in the remaining service time is given by Equation (24).
      (24) $$\begin{array}{cc} \; & P_{n,k}^{\pi }\left(t\right)=e^{-\lambda t}\frac{{\left(\lambda t\right)}^{k}}{k!}\cdot P_{n}^{\pi }\left(t\right)\\ & \mathrm{where},\;n\;=\;\left[0,N\right]\;\mathrm{and}\;k\;=\;\left[0,N-1\right]. \end{array}$$
      Similar to Equation (21), the probability ($P_{n,k}^{\Pi }$) that, at an arbitrary time instant, there are n packets in the system and that during the remaining time of the service k packets have arrived can be calculated as Equation (25).
      (25) $$\begin{array}{cc} \; & P_{n,k}^{\Pi }=\int_{0}^{\infty }P_{n,k}^{\pi }\left(t\right)dt\\ & \;\;\;\;\;\;\;=\frac{1}{\lambda T_{FP}}\sum_{l=1}^{n}\left({\bar{p}}_{l-1}-{\bar{p}}_{0}\cdot B_{l-1}\cdot \bar{e}\right)\cdot \left[\frac{{\left(\lambda \cdot E\left[S\right]\right)}^{n-l+k+1}\cdot {\left(-1\right)}^{n-l}}{\left(n-l)!\cdot k!\cdot (n-l+k+1\right)}\right].\\ & \mathrm{and}\;\\ & P_{N,k}^{\Pi }=\frac{\sum_{l=1}^{N}{\bar{p}}_{l}\cdot \bar{e}}{\lambda T_{FP}}\cdot \left[1-e^{-\lambda \cdot E[S]}\cdot \sum_{i=0}^{k}\frac{{\left(\lambda \cdot E\left[S\right]\right)}^{i}}{(i)!}\right]-\sum_{n=1}^{N-1}P_{n,k}^{\pi }. \end{array}$$
      The average waiting time of a packet that arrives at an arbitrary time instant in a service period is then given by Equation (26).
      (26) $$\begin{array}{cc} \; & T_{n,k}^{\Pi }=\int_{0}^{\infty }t\cdot P_{n,k}^{\pi }\left(t\right)dt\\ & \;\;\;\;\;\;\;=\frac{\sum_{l=1}^{N}\left({\bar{p}}_{l}\cdot \bar{e}\right)}{\lambda T_{FP}}\cdot \left[\frac{{\left(\lambda \cdot E\left[S\right]\right)}^{n-l+k+1}\cdot {\left(-1\right)}^{n-l}\cdot E\left[S\right]}{\left(n-l)!\cdot k!\cdot (n-l+k+2\right)}\right].\\ & \mathrm{and}\;\\ & T_{N,k}^{\pi }=\frac{\sum_{l=1}^{N}{\bar{p}}_{l}\cdot \bar{e}\cdot \left(k+1\right)}{\lambda^{2}\cdot T_{FP}}\cdot \left[1-e^{-\lambda \cdot E[S]}\cdot \sum_{i=0}^{k+1}\frac{{\left(\lambda \cdot E\left[S\right]\right)}^{i}}{(i)!}\right]-\sum_{n=0}^{N-1}T_{n,k}^{\pi }. \end{array}$$
  - Between this inspection instant and the start of the transmission of the packet: Let $T_{n,k}^{s}$ be the mean waiting time of a packet in the FQ at the *end* of a service that will be served and that has n packets (maximum N−2) ahead and k packets (maximum N−n−2) behind is given by Equation (27).
    (27) $$\begin{array}{cc} \; & T_{0,k}^{s}=0,\;\;\;\;\;\;\;\;\;\;\mathrm{for}\;k=[0,N-2]\\ & T_{1,k}^{s}=\sum_{j=0}^{N-k-2}\left(T_{0,k+j}^{s}+E\left[S\right]\cdot \frac{{\left(\lambda \cdot E\left[S\right]\right)}^{j}}{j!}\cdot e^{-\lambda \cdot E[S]}\right),\;\;\;\;\;\;\;\;\;\;\mathrm{for}\;k\;=\;[0,\;N-3]\\ & T_{n,k}^{s}=\sum_{j=0}^{N-n-k-1}\left(T_{n-1,k+j}^{s}+E\left[S\right]\cdot \frac{{\left(\lambda \cdot E\left[S\right]\right)}^{j}}{j!}\cdot e^{-\lambda \cdot E[S]}\right)+\\ & \;\;\;\;\;\;\;\sum_{j=N-n-k}^{N-k-2}\left(T_{N-k-2-j,k+j}^{s}+E\left[S\right]\cdot \frac{{\left(\lambda \cdot E\left[S\right]\right)}^{j}}{j!}\cdot e^{-\lambda \cdot E[S]}\right),\;\mathrm{for}\;k\;=\;[0,\;N-n-2]\\ & \mathrm{and}\;n\;=\;[2,N-2]. \end{array}$$
    Similar to Equation (27), the mean waiting time of a packet in the FQ at the end of a vacation period $T_{n,k}^{v}$ that will be served and has n packets ahead (maximum N−1) and k packets (maximum N−n−1) behind can be calculated.
  The total waiting time of a packet arriving at an arbitrary instant at the FN or average DL latency, served eventually in a system using the push-out strategy, is given by Equation (28).
  (28) $$\begin{array}{cc} T_{PO} & =\sum_{k=0}^{N-1}\left(T_{0,k}^{\Omega }+P_{0,k}^{\Omega }\cdot T_{0,k}^{v}\right)+\sum_{n=1}^{N-1}[\sum_{k=0}^{N-n-1}\left(T_{n,k}^{\Omega }+P_{n,k}^{\Omega }\cdot T_{n,k}^{v}\right)+\\ & \sum_{k=N-n}^{N-1}\left(T_{N,k}^{\Omega }+P_{N,k}^{\Omega }\cdot T_{N-k-1,k}^{v}\right)]\;+\;\sum_{k=0}^{N-2}\left(T_{1,k}^{\Pi }+P_{1,k}^{\Pi }\cdot T_{0,k}^{s}\right)+\\ & \sum_{n=2}^{N-1}\left[\sum_{k=0}^{N-n-1}\left(T_{n,k}^{\Pi }+P_{n,k}^{\Pi }\cdot T_{n-1,k}^{s}\right)+\sum_{k=N-n}^{N-2}\left(T_{N,k}^{\Pi }+P_{N,k}^{\Pi }\cdot T_{N-k-2,k}^{s}\right)\right] \end{array}$$

## 6. Evaluation

This section presents the evaluation of the DL latency and PDR results using the analytical model and the simulator. Firstly, the description of the simulation setup is presented. This is followed by comparing the results of our analytical model and the simulator. The model is implemented in Matlab R2020b, while the simulation program is written in Python 3.9.6. Thereafter, these results are analyzed in detail based on parameters such as harvesting power, capacitor size, data arrival rate, and poll interval timer of a batteryless BLE LPN.

Note that the execution time of the model in Matlab scales with the size of the transition matrix. Therefore, if it is known that, at a specified harvesting rate, the capacitor would be unable to recharge itself up to $V_{max}$ and the matrix size can be reduced by assuming a lower value of $V_{max}$. This behaviour can be easily perceivable for low harvesting rates in combination with large capacitor sizes.

### 6.1. Simulation Setup

**Figure 5:**
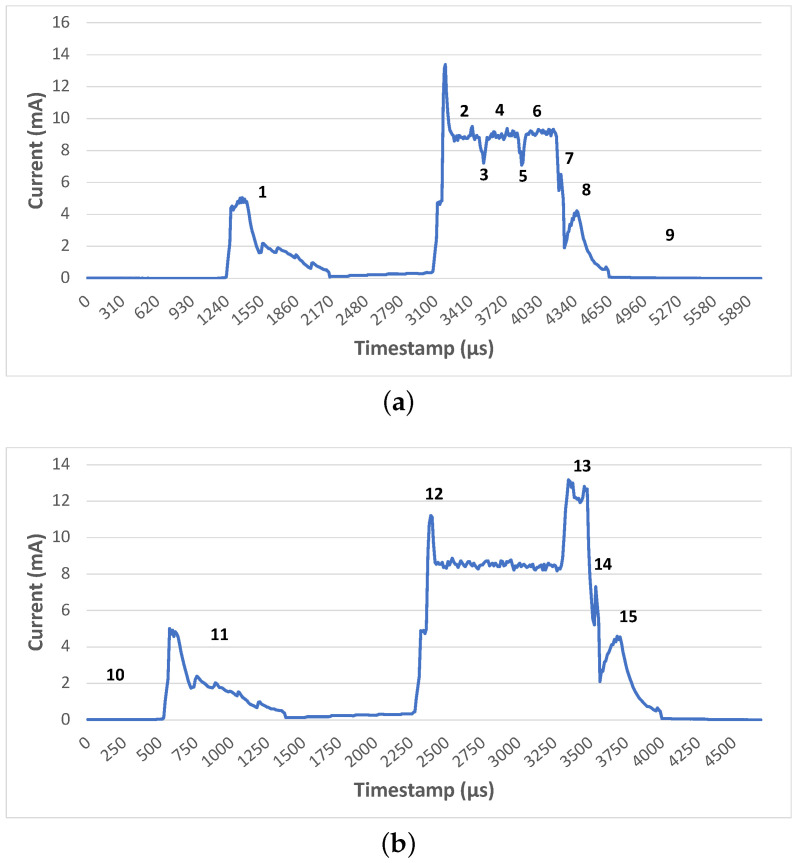
Current consumption of nRF52840 with the state number as defined in Table 1. (**a**) Current consumption during FP; (**b**) Current consumption during Scan.

We implemented an event-based simulator to imitate the friendship communication mechanism of the batteryless LPNs as proposed in [7]. The simulator follows an event-based packet scheduling and is capable of reproducing the BLE radio activities, such as sending FP messages and FQ buffered data packets. It also uses the same capacitor model to imitate the capacitor behaviour, as mentioned in Section 4. Each experiment of the simulator is run until a total of 25,000 packets have been generated according to a Poisson arrival process. To request and receive a buffered data packet, the LPN follows the sequence of states listed in Table 1, in the order from top to bottom. The table also mentions the execution time and the current consumption of the corresponding states of the LPNs. These time and current consumption values can be used as Δt and $I_{L}\left(X\right)$ to calculate the capacitor’s voltage at any time using Equation (1). The time and the current consumption were measured at 1.8 V supply voltage on the Nordic nRF52840 BLE devkit [22] using the Nordic Power Profiler Kit II [23] while running the developed LPN application. All the states of the FP and receiving message mentioned in Table 1 can be seen in Figure 5.

The parameters listed in Table 2 are used to evaluate the model. As such, the values of $V_{cutoff}$ and $V_{max}$ are taken considering one of the configurations of a representative off-the-shelf power management unit [24]. The following performance metrics are considered in the comparison:

- Packet delivery ratio (PDR): The ratio of packets successfully received by the LPN compared to the total reaching the FQ.
- Downlink packet latency: The average latency to receive a packet by the LPN from the time it arrives at the FQ.

### 6.2. Model Validation

The model is validated using the simulation and the emulator. We ran a total of 5640 test cases to compare the results of the analytical model and the simulation. The test cases encompass all possible parameter combinations listed in Table 2. Whereas, using an emulator, a hardware with a lack of automation, we manually ran 25 tests.

#### 6.2.1. Using Simulation

**Figure 6:**
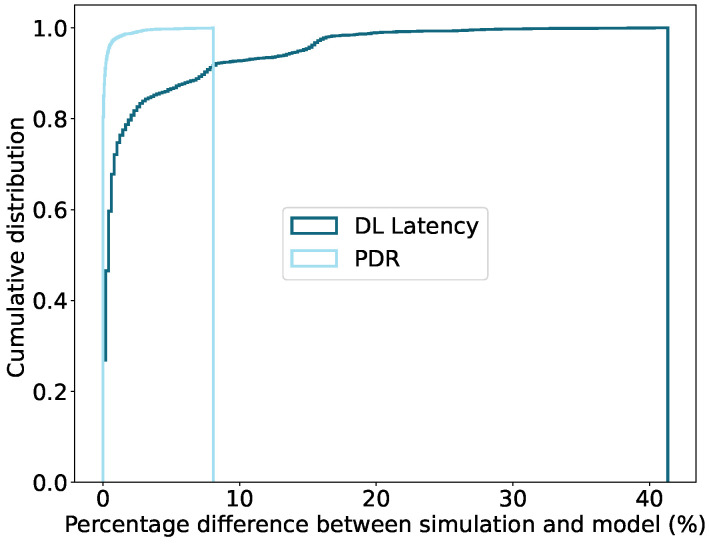
Cumulative distribution of difference in model and simulation results for both the DL latency and PDR metrics.

The harvesting power is considered for harvesting techniques, which are low indoor light or human body temperature, that generate up to 1 mW. To avoid longer latency delay, the poll interval is considered only up to 10 times the data rate. These test cases only analyze DL data. The cumulative distribution shown in Figure 6 presents the relative deviation in PDR and DL latency between the simulation and the model results. We also simulate the device for the large power (more than 1 mW), generating harvesting techniques such as industrial temperature, direct sunlight, or mechanical movement. However, the result obtained with such harvesting techniques would result in 100% PDR with the lowest possible DL latency. Therefore, we exclude these test cases from the result analysis.

There is a mean difference of 2.23% and 0.099% in DL latency and PDR, respectively, found in a run of all the test cases. A high relative difference in PDR value (≥2%) is observed when the PI is set to a relatively larger value than the data arrival rate. For these cases, the relative difference in latency is around 13%. Moreover, the large relative deviation in latency value (≥15%) is also observed for the low $P_{h}$ when the PDR is less than 100%. However, the deviation in latency is higher for the lowest FQ sizes (8 or less). For such cases, the deviation in PDR is only 0.58%.

There are a total of 92.73% and 100% test cases for DL latency and PDR, respectively, with a percentage difference less than 10%. By analyzing the results for the PDR and DL latency of the model and simulation, it is evident that the model’s accuracy is high. However, solving the model is computationally complex for large FQ sizes (32 or more), and the relative mean difference in the DL latency test cases for the lowest FQ size (2 packets) is higher compared to larger queue sizes.

#### 6.2.2. Using Emulator

**Figure 7:**
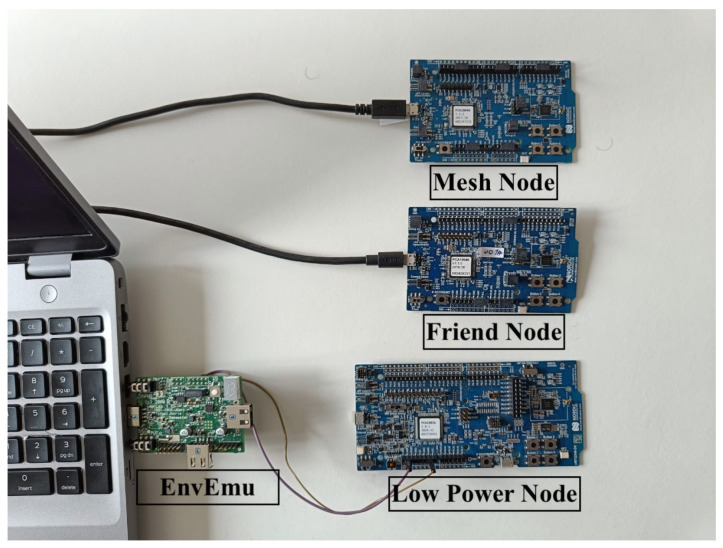
EnvEmu setup.

We use an energy-harvesting emulator called Environment Emulator (EnvEmu) [25], which is a stripped-down version of the TMote Sky. It is connected to the Nordic nRF52840 development board that runs the BLE LPN application, as shown in Figure 7. The EnvEmu is powered by USB and provides a variable input voltage between 1.5 and 3.3 V to Nordic BLE LPN via its VDD-GND pin. The output voltage is based on the emulation of a virtual capacitor and energy harvesting source, which can both be dynamically configured in the EnvEmu via simple commands. An instruction manual for the EnvEmu using Linux can be found in [26]. Two more Nordic nRF52832 development boards powered by USB are also part of the BLE mesh network, of which one acts as an FN and another generates DL packets. The FN temporarily accumulates the incoming DL data from the mesh node, which the batteryless LPN polls.

**Figure 8:**
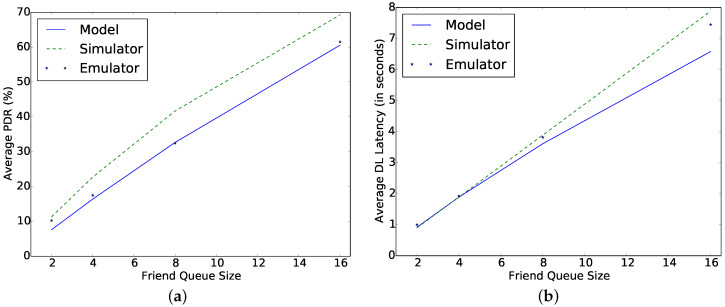
Comparing emulator, simulator, and model for different FQ at $\mu_{p}$ = 1 s, $T_{PI}$ = 30 s, *C* = 1 mF, $P_{h}$ = 0.1 mW, $T_{AT}$ = 5 ms. (**a**) Average packet delivery ratio; (**b**) DL latency.

Figure 8 shows the comparison of the model and emulator for different FQs. It can be observed that the results are similar. The mean difference in the result of the emulator and model is 1.22% and 0.3% in PDR and DL latency, respectively.

### 6.3. Result Analysis Based on Analytical Model and Simulation

**Figure 9:**
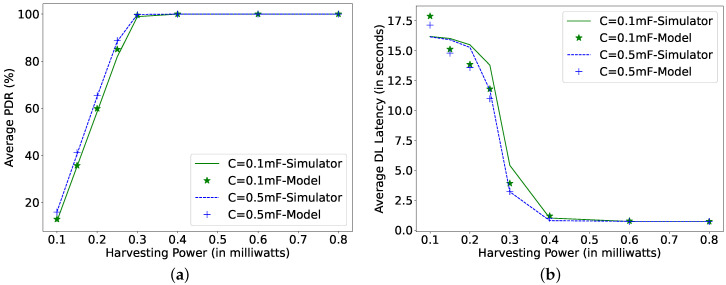
Comparing simulator and model for different harvesting powers at $\mu_{p}$ = 1 s, $T_{PI}$ = 1 s, *N* = 16, $T_{AT}$ = 0 ms. (**a**) Average packet delivery ratio; (**b**) DL latency.

**Figure 10:**
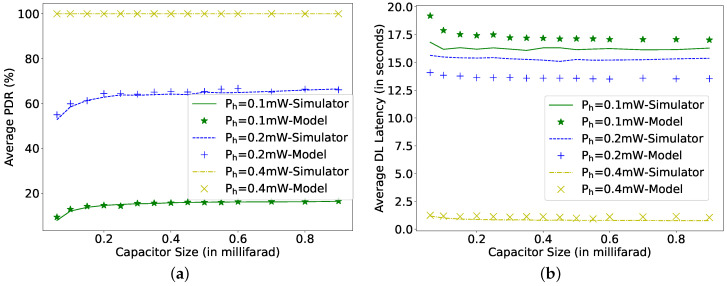
Comparing simulator and model for different capacitor sizes and harvesting power at $\mu_{p}$ = 1 s, $T_{PI}$ = 1 s, *N* = 16, $T_{AT}$ = 0 ms. (**a**) Average packet delivery ratio; (**b**) DL latency.

**Figure 11:**
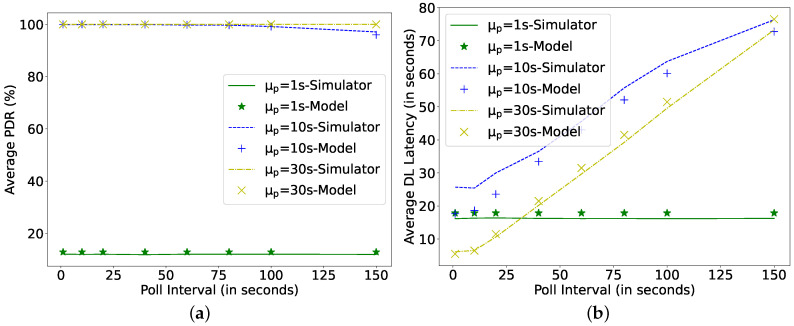
Comparing simulator and model for different Poll Intervals at C = 0.1 mF, $P_{h}$ = 0.1 mW, *N* = 16, $T_{AT}$ = 0 ms. (**a**) Average packet delivery ratio; (**b**) DL latency.

Figure 9, Figure 10 and Figure 11 plot the results of the analytical model and the simulation, showing the effect of all the dependent parameters. It is clear that the analytical model and simulation follow the same behaviour, and the results match closely in all the plotted graphs. It can be observed from Figure 9 that, as the harvesting power increases, the average PDR and DL latency improve until they reach their corresponding maximum achievable values.

The improvement in the PDR and DL latency accelerate faster for low harvesting power until the PDR crosses 80% (0.25 mW in Figure 9a,b). Further, an increase in the harvesting power improves the DL latency at a higher rate than the PDR because, at such harvesting power, the capacitor can recharge faster again to $V_{th}^{FP}$ to receive packets. As soon as the PDR and DL latency reach their corresponding best achievable values, an increase in the harvesting power does not affect the results because the capacitor voltage never drops below $V_{th}^{FP}$, and therefore, the node does not need to go into SLEEP mode to recharge its capacitor. For an arrival rate of 1 s, this harvesting power point is 0.6 mW, as shown in Figure 9. With the decrease in arrival rate, the capacitor gets more time to recharge itself. Therefore, for an arrival rate of 10 s, the mentioned harvesting power point value reduces to 0.3 mW (not shown in Figures). The size of the capacitor also plays a vital role in achieving the desired performance. As a larger capacitor can store more energy, the PDR for the 0.5 mF capacitor is higher than for 0.1 mF. However, the increase of the capacitor size also affects its charging time. Therefore, the performance does not improve much with the increase in the capacitor size after a certain optimal capacitor size for each specific harvesting power. There is an optimal capacitor size for each set of parameters. It can be observed from Figure 10b that, at a harvesting power of 0.1 mW, the optimal capacitor size, where the DL latency is lowest, is 0.35 mF, and for 0.2 mW of harvested power, it is 0.45 mF. An increase in the capacitor size higher than 0.5 mF shows reduced growth in the PDR (harvesting power 0.1 and 0.2 mW). Additionally, when the PDR is 100%, the increase in the capacitor size would improve the DL latency (yellow line at $P_{h}$ 0.4 mW), but not substantially.

Figure 11 shows that, for a fixed harvesting power (0.1 mW), with the decrease in Poisson arrival rate for downlink packets, the PDR can be improved to 100%. This is because the capacitor gets ample time to charge itself before the subsequent transmission. However, the achieved latency can increase with the increase in the poll interval timer as the node has to wait for this PI once it receives the FU. Therefore, the higher the PI, the longer the arriving packets wait in the FQ to be polled. As such, when the data arrival rate is 10 s, some packets are also being dropped for large PI (150 s). Although frequent polling consumes additional energy, the considered harvesting power (0.1 mW) is sufficient to support a PI as low as 1 s without negatively affecting PDR. Moreover, the DL latency at the arrival rate of 1 s is not affected by the increase in PI, because there are always packets waiting in the FQ to be polled, and the LPN never receives an FU message.

**Figure 12:**
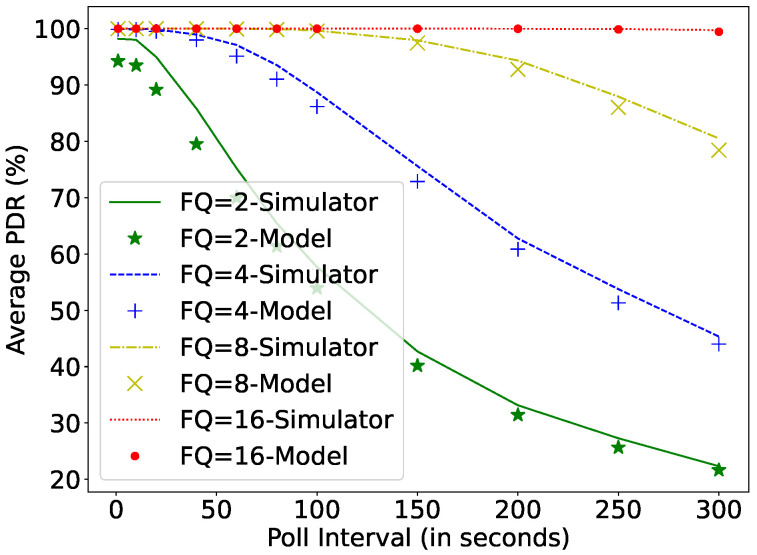
Comparing simulator and model for different poll intervals and FQ at C = 0.1 mF, $P_{h}$ = 0.1 mW, $\mu_{p}$ = 30 s, $T_{AT}$ = 0 ms.

If the size of the FQ decreases, then the number of dropped packets increases for higher PI. This can be observed in Figure 12. The smaller the FQ, the lower the PDR. Another interesting parameter of the LPN is the AT, which also affects the power consumption and DL latency, as the LPN needs to actively listen during the configured time. With the increase in AT, the LPN wastes energy in idle listening on the incoming channels.

**Figure 13:**
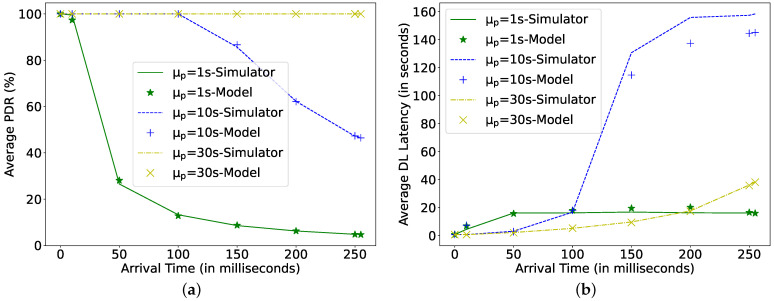
Comparing simulator and model for different arrival times (AT) at C = 4 mF, $P_{h}$ = 1 mW, *N* = 16, $T_{PI}$ = 1 s. (**a**) Average packet delivery ratio; (**b**) DL latency.

Figure 13 shows its effect on DL latency and PDR. It can be observed that a slight increase in AT can drastically affect the PDR and, therefore, the DL latency. However, when the data arrival rate is low, the LPN has sufficient time to recharge its capacitor to maintain a PDR of 100%, but the increase in AT affects the DL latency exponentially. As seen for the Poisson arrival rate of 30 s, the latency increases 60-fold from 0.65 s at 0 s AT to 38.43 s at AT of 255 ms. At a high Poisson arrival rate (i.e., 1 s), the impact of the AT is higher because the LPN has less time to charge its capacitor and needs to receive more packets compared to lower Poisson data arrival rates. Increasing the AT further affects the performance marginally. Therefore, AT should be configured as small as possible to achieve high LPN performance. However, a small AT value provides less time to the FN to send a response to the LPN, which in turn affects the scalability (i.e., it causes missed response opportunities when multiple LPNs expect a response simultaneously).

**Figure 14:**
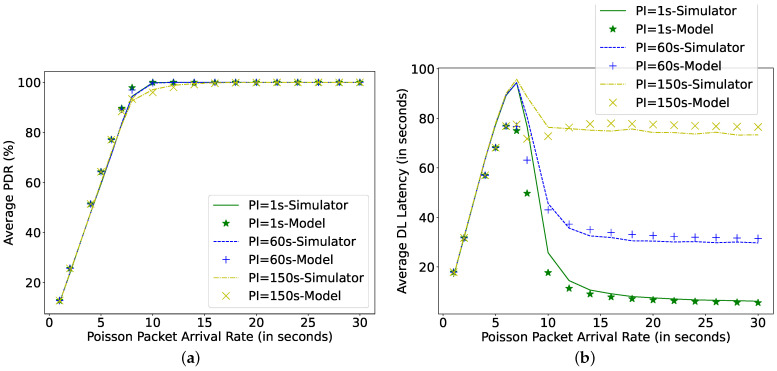
Comparing simulator and model for different packet arrival rate at C = 0.1 mF, $P_{h}$ = 0.1 mW, *N* = 16. (**a**) Average packet delivery ratio; (**b**) DL latency.

Furthermore, from Figure 14, it can be observed that an LPN having a capacitor of 0.1 mF and a harvesting power of 0.1 mW can receive almost all the data packets when the arrival rate is 10 s for poll interval timers lower than 60 s. Further, decreasing the data arrival rate (from 7 s to 30 s) would improve the latency because fewer packets are entering into the FQ and the capacitor gets more time to recharge itself. Similarly, it improves the PDR when the data arrival rate decreases from 1 s to 7 s. However, the DL latency increases in this interval because with the decrease in data interval fewer packets are entered into the FQ and thus wait longer to be polled.

## 7. Use-Case: Logistic Management

**Figure 15:**
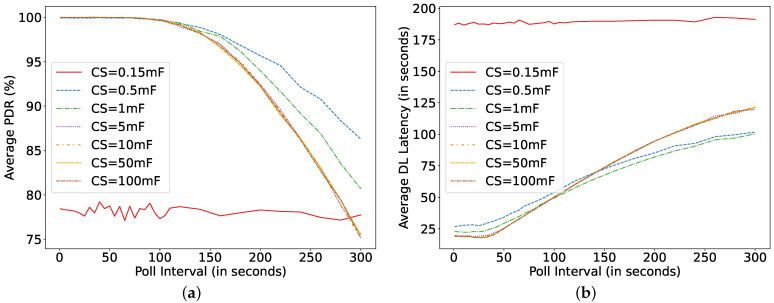
Asset monitoring at DR = 30 s, $P_{h}$ = 0.1 mW, *N* = 8, $T_{AT}$ = 5 ms. (**a**) Average packet delivery ratio; (**b**) DL latency.

This section presents the use of the proposed model to derive optimal parameters for a batteryless BLE node, considering the IoT use-case of the logistics process. Logistics is one of the major industries adopted by IoT at scale. IoT applications can facilitate asset monitoring in warehouses or shops. Instead of checking the assets manually, they can be connected via batteryless BLE devices that allow effortless tracking of each item. This batteryless device can listen to incoming queries to respond about its availability or transmit sensor data. Consider a use-case where a server queries for the asset availability every 30 s and the batteryless node can harvest 100 μW. The model can be used to find the optimal BLE batteryless LPN configuration, including the capacitor size and poll interval. Assuming a friend queue size of eight packets and an arrival time of 5 min, the model provides the result as shown in Figure 15. It can be observed that the PDR is 100% for lower PI values and the capacitor which are greater than equal to 0.5 mF. However, DL latency is lower for a capacitor of 5 mF or more and does not vary much. Therefore, considering the charging and discharging time of a capacitor, we should select the lowest possible capacitor, which, for this use-case, can be considered as 5 mF. Additionally, the DL latency is lowest (18.27 s) at a PI equal to 20 s.

**Figure 16:**
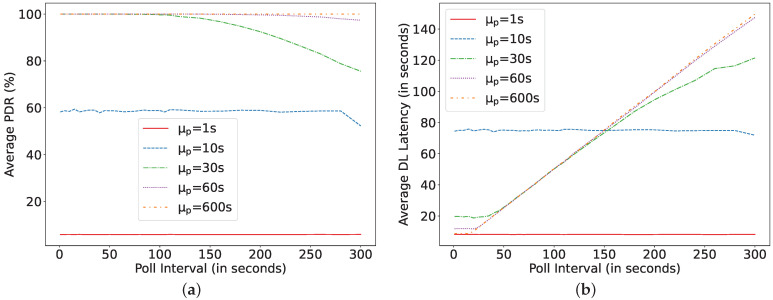
Asset monitoring at C = 5 mF, $P_{h}$ = 0.1 mW, *N* = 8, $T_{AT}$ = 5 ms for different packet arrival rate. (**a**) Average packet delivery ratio; (**b**) DL latency.

Moreover, if the packet arrival rate from the server changes, the model can be used to determine the new expected performance of this batteryless LPN that harvests 100 μW and can be configured with a capacitor of 5 mF and polling at 20 s. The result is shown in Figure 16. It can be observed that, at a high arrival rate (1 and 10 s), many packets drop, whereas, lowering the packet arrival rate (60 and 600 s) improves the DL latency up to 55% without affecting the PDR.

## 8. Conclusions

In this paper, we presented an analytical model to calculate the DL latency and PDR for a batteryless BLE LPN operating on power harvested from natural or artificial indoor light. The model’s accuracy was compared with Python-based simulation results for various parameters (i.e., capacitor size, harvesting power, data arrival rate, poll interval timer, arrival time, and friend queue size). The results derived from these two methods showed an average deviation of 2.23% in the results of DL latency and 0.09% in PDR. The graphs of different parameters showed diverse behaviour for DL latency and PDR, whereas the model and simulation show the same pattern.

The results showed that a harvesting power of 0.6 mW can maintains a DL packet inter-arrival rate as low as 1s, and at 0.3 mW, the LPN can receive all the packets arriving at a rate of 10 s to an FQ of size 16. The smallest optimal capacitor size should be chosen depending on the available minimum harvesting power so that it can be recharged rapidly. The poll interval timer affects the DL latency linearly if the PDR is 100%. Therefore, it is important to choose the lowest possible poll interval timer that can be supported by the minimum harvesting power. If the harvesting power varies over time, the poll interval timer should be dynamically adapted. The external factors, such as Friend Queue size, also affect the performance of the batteryless LPN. A small queue size increases the packet loss. In our experiments, a queue size of 16 packets was enough to maintain 100% PDR, for polling intervals as high as 300 seconds at a downlink packet arrival rate of 30 seconds. Moreover, the AT parameter should be configured to be as small as possible so that the LPN does not lose energy in idle listening on the incoming channels. However, a very low AT may negatively affect scalability. For a harvesting power of 1mW, an AT up to 100 ms could be supported without a decrease in PDR.

## 9. Discussion and Future Work

There are several future research directions. In our model, we have considered a power management system that supplies variable voltage directly from the capacitor. However, more advanced power management solutions exist that equalize the supply voltage and only provide the device with power if the capacitor voltage is within certain minimum and maximum bounds [27]. Such solutions offer more consistent and stable performance, as then the power consumption for each radio state would be stable with a stable voltage supply. Most of the existing works in the batteryless domain do not consider this [28,29,30,31]. Therefore, in the future, we will explore adapting the batteryless circuit model for such power management solutions. However, in this case, an additional circuit would be required to read the capacitor voltage. CusTARD uses an Analog-to-digital (ADC) pin to read the capacitor voltage [32]. The ADC pin can read values in a range that is usually lower than the capacitor voltage. Therefore, a voltage divider would be required, which increases the power consumption of the device (several tens of μW) and therefore also affects the performance in terms of PDR and DL data latency for different harvesting powers. This could be incorporated into our model by changing Equation (1) and adding the current consumption of the additional circuit to the load current. Other modules used for voltage reading are Torpor Logic and Power switch [33], which can consume up to 2 μW rather than the assumed ultra-low-power comparator that consumes power in the order of pico-watts. As such, we need to perform measurements using real hardware to incorporate this power consumption into the model, as the hardware circuitry to enable measuring the capacitor voltage can impact the results.

The harvesting architecture considered is harvest-store-use. However, there is another architecture ‘harvest-use’ that eliminates the use of capacitors for energy storage [34]. This architecture can reduce the device cost and efficiency. A prototype of such an architecture is presented by Lee et al. [35]. Our model can be extended to a storage-less architecture. In this case, the performance is mainly governed by the harvesting power without a capacitor. Therefore, the stochastic process would consider the number of packets in the FQ and the harvesting source power. A certain minimum harvesting power is then required before and during execution of each task. Therefore, we need another additional circuitry, such as a sense resistor to measure the power of the harvesting source, which further increases the power demand of the batteryless device. Therefore, the requirements and performance of this architecture would be different.

Additionally, we aim to extend the analytical model to consider a variable harvesting power, in contrast to the currently considered constant harvesting power. This would provide a realistic harvesting model to evaluate harvesting sources. such as solar or wind. The model would consider only the current amount of harvested energy. Such solutions are realized by Zhou et al. [36], Patil et al. [37], and Kumar et al. [38] for investigating wireless sensor networks with energy harvesting. An approach to achieve this is by considering multiple energy chunks that are harvested during different Poisson distributed time slots, where the harvesting power during a slot remains constant [37]. As such, our existing model can be relatively easily extended to such a scenario by applying it to different harvesting power inputs for each variable-length time slot.

Finally, the increased CPU utilization due to servicing multiple LPNs may slow down the LPN’s response and thus affect the DL latency. Therefore, another aspect is to study the interactions and effects of multiple LPNs associated with one FN. To date, there are no works that study this aspect. However, research has been done to study the effect on discovery latency due to the number of scanners [39] or due to interference in the presence of many BLE nodes [40]. With an increasing number of BLE devices, delays of device discovery show exponential growth. Due to interference, the latency can increase up to 400% in a network of 30 devices. This implies that severe contention can occur among multiple BLE devices while considering bi-directional communication using a batteryless device.

## Figures and Tables

**Table 1:** State sequence for a poll request and response of a LPN.

Identifier	Radio State	Time [μs] of the LPN [mA]	Current Cons.	Comments
1	Radio wake-up	2940	0.587	Prepare for poll transmission
2	TX (at 4 dBm)	471	9.09	Tx advertisement packet on channel 37
3	Change channel	29	7.78	Change radio frequency
4	TX (at 4 dBm)	380	9.15	Tx advertisement packet on channel 38
5	Change channel	29	7.57	Change again the radio frequency
6	TX (at 4 dBm)	384	9.16	Tx advertisement packet on channel 39
7	Radio off	34	6.60	Main radio in OFF state
8	Post processing	420	2.17	
9	Cool down	20,820	0.00653	Prepare to switch to the SLEEP state
10	Sleep	RD	0.00896	Node in SLEEP mode
11	Wake-up prescan	1910	1.13	Wake-up for Rx
12	Listen	AT	8.68	Actively Listen for incoming DL packet
13	Scan message	1160 (or 900)	9.64	Rx 24 B FQ data (or 22 B FU)
14	Radio off	389	4.69	Main radio in OFF state
15	Post-processing and Cool down	23,410	0.00619	Set up the sleep timer for the next state and switch to SLEEP state

**Table 2:** Simulation parameters.

Parameters	Symbol	Values/Range
Poisson packet arrival rate	$\mu_{p}$	[1, 10, 30, 60, 120] s
Harvesting power	$P_{h}$	[0.1, 0.15, 0.25, 0.3, 0.35, 0.2, 0.4, 0.6, 0.8, 1] mW
Capacitor size	*C*	[0.1, 0.2, 0.3, 0.4, 0.5, 1] mF
Poll interval	$T_{PI}$	[1, 10, 20, 40, 60, 80, 100, 150, 200, 250, 300, 400, 500, 600, 800, 1000] s
Turn-off voltage	$V_{cutoff}$	2.8 V
Max operating voltage	$V_{max}$	4.5 V
Friend Queue size	*N*	[8, 16] packets
Receive Delay	$T_{RD}$	255 ms
Arrival time (max up to $T_{RW}$)	$T_{AT}$	0 ms
Tx power level		0 dBm

## Data Availability

Not applicable.

## Appendix A. Parameters Used in the Model

Table A1 summarizes all the important parameters’ symbol with their definition.

**Table A1:** Parameters Used in the Markov Chain Model.

Symbol	Meaning
λ	Poisson data arrival rate
N	FQ size
U	Voltage Vector
$S_{i}$	Duration of service when U = i
$W_{i}$	Duration of vacation when U = i
$V_{i}^{S}$	Voltage at next poll instance when U = i at the start of a service
$V_{i}^{W}$	Voltage at next poll instance when U = i at the start of a vacation
$E[S_{i}]$	Average duration of $S_{i}$
$E[W_{i}]$	Average duration of $W_{i}$
$\bar{p}$	Probability distribution vector of Markov Chain
${\bar{p}}_{i}$	Probability distribution of the voltage equals i
(${\bar{p}}_{k}{)}_{i}$	Probability distribution of the voltage equals i if the queue length is k
$B_{i}$	Transitions starting from an empty system and ending up with i packets in FQ at the next FP
$A_{i}$	Transitions starting from a non-empty system and ending up with i packets in FQ at the next FP
$O_{n}$	System occupancy when n number of packets are present in FQ
$O_{DT}$	Average system occupancy in drop-tail system
$O_{PO}$	Average system occupancy in push-out system
$P_{k,i}^{S}$	Probability that k packets arrive at service time starting from U = i
$P_{k,i}^{W}$	Probability that k packets arrive at vacation time starting from U = i
$P_{n}^{\omega }\left(t\right)$	Probability that the packet arrives in a vacation period and n packets are present in the FQ at time t
$P_{n,k}^{\omega }\left(t\right)$	Probability that there are packet arrives in a vacation period and n packets are present in the FQ at time t and k packets arrive in the remaining vacation time
$P_{n,k}^{\Omega }$	Probability that the packet arrives in a vacation period and n packets are present in the FQ and k packets arrive in the remaining vacation time
$P_{n}^{\pi }\left(t\right)$	Probability that the packet arrives in a service period and n packets are present in the FQ at time t
$P_{n,k}^{\pi }\left(t\right)$	Probability that the packet arrives in a service period and n packets are present in the FQ at time t and k packets arrive in the remaining service time
$P_{n,k}^{\Pi }$	Probability that the packet arrives in a service period and n packets are present in the FQ and k packets arrive in the remaining service time
$\mu_{E[W]}\left(t\right)$	Heaviside function for E[W]
$\mu_{E[S]}\left(t\right)$	Heaviside function for E[S]
$T_{FP}$	Average Time between two consecutive FPs
$T_{DT}$	DL latency in drop-tail system
$T_{PO}$	DL latency in push-out system
$T_{n,k}^{\Omega }$	Average waiting time of a packet that arrives in a vacation period
$T_{n,k}^{\Pi }$	Average waiting time of a packet that arrives in a service period
$T_{n,k}^{s}$	Mean waiting time of a future served packet in the FQ at the end of a service period that has n packets ahead and k packets behind it
$T_{n,k}^{v}$	Mean waiting time of a future served packet in the FQ at the end of a vacation period that has n packets ahead and k packets behind it
